# A Regulatory Circuitry Between Gria2, miR-409, and miR-495 Is Affected by ALS FUS Mutation in ESC-Derived Motor Neurons

**DOI:** 10.1007/s12035-018-0884-4

**Published:** 2018-02-12

**Authors:** Davide Capauto, Alessio Colantoni, Lei Lu, Tiziana Santini, Giovanna Peruzzi, Silvia Biscarini, Mariangela Morlando, Neil A. Shneider, Elisa Caffarelli, Pietro Laneve, Irene Bozzoni

**Affiliations:** 1 Center for Life Nano Science@Sapienza, Istituto Italiano di Tecnologia, Viale Regina Elena 291, 00161 Rome, Italy; 2 Department of Biology and Biotechnology, Sapienza University of Rome, Piazzale Aldo Moro 5, 00185 Rome, Italy; 3 Department of Neurology, Center for Motor Neuron Biology and Disease, Columbia University, 630 West 168th Street, New York City, 10032 NY USA; 4 Present Address: Institute of Molecular Biology and Pathology, CNR, Piazzale Aldo Moro 7, Rome, 00185 Italy; 5 Institute Pasteur Fondazione Cenci-Bolognetti, Sapienza University of Rome, Piazzale Aldo Moro 5, Rome, 00185 Italy

**Keywords:** ALS, FUS, RNA binding proteins, Motor neurons, Gria2, Excitotoxicity, MicroRNAs

## Abstract

Mutations in fused in sarcoma (FUS) cause amyotrophic lateral sclerosis (ALS). FUS is a multifunctional protein involved in the biogenesis and activity of several types of RNAs, and its role in the pathogenesis of ALS may involve both direct effects of disease-associated mutations through gain- and loss-of-function mechanisms and indirect effects due to the cross talk between different classes of FUS-dependent RNAs. To explore how FUS mutations impinge on motor neuron-specific RNA-based circuitries, we performed transcriptome profiling of small and long RNAs of motor neurons (MNs) derived from mouse embryonic stem cells carrying a FUS-P517L knock-in mutation, which is equivalent to human FUS-P525L, associated with a severe and juvenile-onset form of ALS. Combining ontological, predictive and molecular analyses, we found an inverse correlation between several classes of deregulated miRNAs and their corresponding mRNA targets in both homozygous and heterozygous P517L MNs. We validated a circuitry in which the upregulation of miR-409-3p and miR-495-3p, belonging to a brain-specific miRNA subcluster implicated in several neurodevelopmental disorders, produced the downregulation of Gria2, a subunit of the glutamate α‐amino‐3‐hydroxy‐5‐methyl-4-isoxazole propionic acid (AMPA) receptor with a significant role in excitatory neurotransmission. Moreover, we found that FUS was involved in mediating such miRNA repression. Gria2 alteration has been proposed to be implicated in MN degeneration, through disturbance of Ca^2+^ homeostasis, which triggers a cascade of damaging “excitotoxic” events. The molecular cross talk identified highlights a role for FUS in excitotoxicity and in miRNA-dependent regulation of Gria2. This circuitry also proved to be deregulated in heterozygosity, which matches the human condition perfectly.

**Electronic supplementary material:**

The online version of this article (10.1007/s12035-018-0884-4) contains supplementary material, which is available to authorized users.

## Introduction

Amyotrophic lateral sclerosis (ALS) is a fatal neurological disease characterized by the degeneration of motor neurons (MNs) in the brain and spinal cord, which results in progressive weakness and motor dysfunction [1]. Familial ALS (fALS) accounts for about 10% of all cases, the vast majority of which are sporadic in onset (sALS). Mutations found in dozens of genes have been causally associated with fALS, and a significant number of these encode RNA-binding proteins with multiple functions in RNA metabolism [2]. TDP-43 (Tar-DNA binding protein 43 or TARDBP) [3–5] and FUS (fused in sarcoma) [6, 7] were the first two RNA-associated factors to be genetically linked to ALS. Both proteins are predominantly nuclear, but are able to shuttle between the nucleus and cytoplasm [8]. In the case of FUS, more than 50 specific mutations have been found in fALS patients, mainly clustered at its C-terminal nuclear localization signal (NLS) [9]. Defective nuclear import may lead to the loss of FUS nuclear function and/or deregulation of its cytoplasmic activity; however, FUS loss of function alone is not sufficient to cause MN degeneration [10]. Moreover, nuclear gain of toxic function due to the altered interactome of FUS mutants cannot be excluded.

The pleiotropic role of FUS on RNA metabolism suggests the intriguing possibility that ALS is an RNA disorder [11]. Several studies have reported the results of transcriptome analyses in cell lines in which wild-type or mutant FUS are either overexpressed [12] or silenced [13]. Other studies have involved the analysis of tissues (striatum or spinal cord) from FUS-transgenic [14] or FUS-depleted mice [15] or of mixed neural populations derived in vitro from mouse embryonic stem cells (mESCs) treated with anti-FUS siRNAs [16]. More recently, RNA expression was profiled from the brain or spinal cord of homozygous [17] and heterozygous [18] FUS knock-in mice. They carried a FUS allele which lacks exon 15, including the regulatory elements present in the 3′-UTR [18]. These experimental systems lead to almost complete or partial loss of nuclear FUS, but none of them faithfully reproduce the defect(s) found in patients. Moreover, none of these studies explore the possible effects of FUS on the intricate cross talk between mRNAs and microRNAs whose biogenesis and activity are regulated by FUS. To address both issues, we carried out the transcriptome analysis of small and long RNAs in MNs derived in vitro from mESCs carrying the FUS-P517L knock-in mutation, corresponding to the human FUS-P525L allele. This allele is found in patients with a juvenile-onset form of the disease [19] and leads to progressive accumulation of cytoplasmic FUS [20]. In vitro studies have shown that this mis-localization is exacerbated by different types of stress [21]. Using a high-throughput next generation sequencing (NGS) approach, we have identified several deregulated miRNA/mRNA interactions. The one involving Gria2, known to be implicated in ALS neurotoxicity [22], together with miR-409-3p and miR-495-3p, belonging to the miR379-410 cluster, deregulated in several neurological disorders [23], provides a novel link between FUS and ALS pathogenesis. Notably, this circuitry also proved to be deregulated in MNs which are heterozygous for the FUS mutation, which reflects the genetic background of the human pathology. We also made the novel observation that FUS can cooperate with miRNAs by supporting their repression activity on target 3′-UTRs.

## Methods

### Oligonucleotides

Oligonucleotide sequences used in this study are listed in Online resource 1: Table 1.

### Cell Cultures and Treatments

FUS^WT^ or FUS^KO^ or FUS^HOMO^ and FUS^HET^ mESCs were cultured and differentiated into spinal motor neurons (MNs) as described in Wichterle [24] by culturing embryoid bodies (EBs) in ADNFK medium complemented with B27 supplement, retinoic acid (RA), and smoothened agonist (SAG). Further details are found in Online resource 2 (supplementary methods and references).

N2a cells, from ATCC (Cat. No. CCL-131), were cultured in DMEM medium D6546 (Sigma-Aldrich) supplemented with 10% fetal bovine serum (F7524, Sigma-Aldrich), l-glutamine (G7513, Sigma-Aldrich), and penicillin-streptomycin (P0781, Sigma-Aldrich).

### Isolation of Motor neurons by FACS

MNs were resuspended in PBS without Ca^++^Mg^++^, 2.5% horse serum, 0.4% glucose, and DNAse I, containing 2% B27 supplement and sorted for GFP expression using a FACSAria III (Becton Dickinson, BD Biosciences) equipped with a 488-nm laser and FACSDiva software (BD Biosciences version 6.1.3). Analysis was based on FlowJo software (Tree Star). Details are given in supplementary methods.

Cells were replated on 0.01% poly-l-ornithine and 20 μg/ml natural mouse laminin (Sigma-Aldrich)-coated dishes, in motor neuron medium (Neurobasal medium, 2% horse serum, 1% B27, 1% Pen/Step, 0.25% 2-mercaptoethanol, 0.25% Glutamax, 0.025 mM l-glutamic acid) supplemented with 10 ng/ml BDNF, 10 ng/ml GDNF, 10 ng/ml CNTF, 10 ng/ml NT3 from Thermo Fisher, and ROCK inhibitor (20 μM) for the first 48 h.

### Overexpression and Depletion Experiments

Constructs Luc/Gria2: WT Gria2 3′-UTR was PCR-amplified from cDNA generated from sorted MNs with the oligonucleotides NotI-Fw and NotI-Rev and cloned in the psiCheck2 plasmid. The mutant versions were derived from a wild-type construct by the QuikChange II Site-Directed Mutagenesis Kit (Agilent). Luc/Gria2/409, carrying mutations in the miR-495-3p-responsive elements (MREs) for miR-409-3p, was generated using oligonucleotides mut 409-3p (1-4), whereas Luc/Gria2/495, carrying mutations for miR-495-3p, was generated through oligonucleotides mut 495-3p (1-4). The construct Luc/Gria2/409-495 was derived combining both sets of primers.

Plasmids and miR mimics were co-transfected with Lipofectamine 2000 (Thermo Scientific) as described below.

FUS depletion in N2A cells was obtained by overnight transfection of siRNA against FUS (5′-GAGTGGAGGTTATGGTCAA-3′) or scrambled siRNAs (AllStars Negative Control siRNA, 1027281, Qiagen) using Lipofectamine 2000 (Thermo Fisher Scientific) according to the manufacturer’s instructions.

Overexpression of FUS^P525L^ was obtained by transfection of Flag-epB-Puro-TT-derived plasmid as described in [25].

FUS protein was induced by adding Dox (0.2 μg ml^−1^) to the culture medium for 24–48 h.

### Luciferase Assay

N2A cells were plated and co-transfected with psiCheck2 expressing Luc/Gria2 (plasmid: 50 ng/ml of transfection mix) and 20 nM of each miR mimic (specific or scrambled). Forty-eight to 72 h after transfections, cells were lysed and luciferase activity was measured in GloMax-Multi+ Detection System (Promega), using Dual-Luciferase Reporter Assay System (Promega). Luciferase assays were also carried out upon FUS depletion or ectopic expression, as described above.

### Protein Extraction and Western Blot

Whole-cell protein extracts were prepared using RIPA buffer and subjected to western blot analysis with precasted NuPAGE 4–12% Bis-Tris gels and reagents (Life Technologies). The immunoblots were incubated with the following antibodies, diluted in 5% skim milk in TBS-T: FUS/TLS (sc-47711, Santa Cruz, 1:2000), GAPDH (sc-32233, Santa Cruz, 1:2000), and GRIA2 (11994-1-AP, Proteintech, 1:1000). All the images were acquired using the Molecular Imager ChemiDoc XRS+ (Bio-Rad), and the densitometric analyses were performed using the associated Image Lab software (Bio-Rad).

### RNA Preparation and Analysis

Total RNA from cells was extracted with the Quick-RNA MiniPrep (Zymo Research) and retrotranscribed with SuperScript VILO (Life Technologies) or miScript II RT (Qiagen) for mRNAs and microRNAs, respectively. Real-time qRT-PCR analysis was performed with PowerUP SYBR Green Master Mix (Life Technologies) for mRNAs or SYBR Green PCR Master Mix (Qiagen) for microRNAs.

The internal control for mRNA analysis is the housekeeping gene Atp5o (ATP synthase, H+ transporting, mitochondrial F1 complex, O subunit). For miRNA analysis, the internal control was U6 snRNA.

### RNA-Seq and Bioinformatics Analysis

TruSeq Stranded Total RNA Library Prep Kit with Ribo-Zero treatment (Illumina) was used to obtain sequencing libraries from total RNA extracted from sorted GFP(+) FUS^WT^, FUS^HOMO^, and FUS^KO^ MNs. The sequencing reaction, which produced 100 nucleotides-long paired-end reads, was performed on an Illumina HiSeq 2500 Sequencing system.

Alignment of reads to mouse genome and transcriptome was performed using TopHat2 software [26].

Cuffdiff 2 was employed for gene- and transcript-level quantification and for differential expression analysis [27].

### Small RNA-Seq

Small RNA libraries were generated from total RNA extracted from sorted GFP(+) FUS^WT^, FUS^HOMO^, and FUS^KO^ MNs using TruSeq Small RNA Library Preparation Kit. Fifty-nucleotide single-end sequencing was performed on an Illumina HiSeq 2500 Sequencing system. Bowtie [28] was used to align reads to the sequence of canonical microRNAs and their putative isoforms. Full quantile normalized read counts were provided to edgeR [29] for differential expression analysis. DIANA-microT web server [30] was used to retrieve information on miRNA-target interactions predicted by microT-CDS software, using a threshold for the target prediction score equal to 0.7.

### CLIP-Seq Data Reanalysis

Raw reads from the FUS HITS-CLIP experiment conducted by Lagier-Tourenne and co-workers [15] on wild-type whole mouse brain were downloaded from GEO and reanalyzed following a pipeline similar to that described in Errichelli [25]. This dataset was selected because of the significant inter-replica peak consistency. Transcripts bound by FUS in the 3′-UTR were found by intersecting the genomic coordinates of these untranslated regions with those of FUS peaks using bedtools intersect [31].

### Immunofluorescence

Cells were cultured on poly-l-ornithine/laminin-pre-coated glass coverslips and then fixed in 4% paraformaldehyde in PBS for 20 min at 4 °C. Double immunostainings were performed sequentially as described above [32]. In brief, cells were permeabilized with Triton 0.3% (10 min, RT), blocked with 2% BSA/5% goat serum in PBS (20 min, RT), and then incubated with anti-FUS antibody (sc-47711, Santa Cruz) 1:100 in 1% BSA/1% goat serum/PBS (ON, at 4 °C). Target detection was performed by goat anti-mouse Cy3 conjugated antibody (Jackson ImmunoResearch, 115-165-003; 1:300). After washing, cells were blocked with 10% normal mouse serum/1% goat serum/1% BSA (30 min, RT). Sequential labeling with a second primary antibody (anti-Islet-1/2 39.4D5, DSHB; 1:50) and detection with donkey anti-mouse Alexa Fluor 647 (Invitrogen A-31571; 1:100) was carried out. An endogenous GFP pattern was detected through expression from the HB9::GFP cassette. Nuclei were labeled with DAPI (Sigma, D9542; 1 μg/ml/PBS). Coverslips were mounted using ProLong Diamond Antifade Mountant (Thermo Fischer Scientific P-36961).

### Confocal Microscopy and Post-Acquisition Analysis

Samples were imaged on a confocal laser scanning microscope (FluoView FV10i Olympus) by using a ×60 water immersion objective (NA 1.35). Images were captured at depth intervals of 0.3 μm and a resolution of 1024 × 1024 pixels. Laser intensity was set for each channel for optimal visualization of fluorescent labeling, and kept constant for each acquisition. All Z-stacks were processed with ImageJ/FIJI software and merged in Z-projection 16-bit color images. The intensity threshold was adjusted considering the signal of cells incubated without primary antibodies as background. Line scan analysis was performed with FIJI to plot the fluorescence intensity values along a selected line (intensity vs distance) after subtracting the background values obtained in a region next to cells that did not show fluorescence. The fluorescence intensity values obtained from the cytoplasmic region of the line scan analysis were then used to calculate the mean value +/− SEM.

### Statistical Analysis

Results are expressed as means +/− SEM from biological triplicates. Statistical differences were analyzed by using two-tailed Student’s *t* test. A *p* value < 0.05 was considered as statistically significant: *p < 0.05, **p < 0.01, ***p < 0.001.

#### Data Availability

RNA sequencing raw data have been deposited at Gene Expression Omnibus (GSE 101097).

## Results

### In Vitro Differentiation and FACS Purification of Spinal MNs from mESCs

**Fig. 1:**
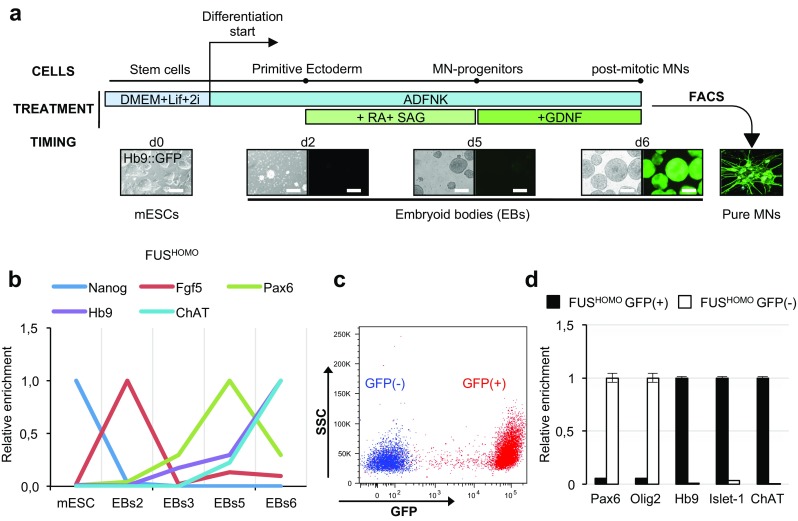
In vitro differentiation of mESC into spinal MNs. **a.** Schematic overview of the differentiation protocol: cell types, experimental treatments, and timing are indicated (d = day, RA = retinoic acid, SAG = smoothened agonist, FACS = fluorescence-activated cell sorting, GDNF = glial cell-derived neurotrophic factor, ESCs = embryonic stem cells, EBs = embryoid bodies, MNs = motor neurons). See text for details. Scale bar: 200 μm. **b** qRT-PCR profiling of stemness, primitive ectoderm, and neural/MN markers along differentiation of FUS^HOMO^ mESCs to MNs. Cell types/differentiation days (2 to 6) are indicated on the x-axis. For each marker analyzed (indicated above), the expression peak is set as 1. Results are expressed in arbitrary units, relative to Atp5o as internal standard. **c** mESCs differentiated to MNs were isolated by FACS based on GFP expression level. Upon sorting, FUS^HOMO^ GFP(−) (blue) and GFP(+) (red) cells were checked for purity. The figure shows the overlay of the purified populations. **d** qRT-PCR analysis of neural/MN progenitors and MN markers in sorted FUS^HOMO^ GFP(+) (black bars) and GFP(−) cell populations (white bars). For each marker analyzed (indicated below), the expression peak is set as 1. Results are expressed in arbitrary units, relative to Atp5o as internal standard

Based on the protocol described in Wichterle [24, 33] (Fig. 1a), we differentiated in vitro spinal MNs from mESCs derived from a homozygous knock-in mouse (NAS, in preparation) carrying the FUS-P517L allele (FUS^HOMO^). The homozygous condition was initially selected in order to analyze the effects of the mutation in the absence of any wild-type protein.

MN differentiation was monitored by following the expression of several markers (Fig. 1b). The stemness-related factor NANOG [34] was expressed only in ESCs, and it was switched off before the appearance of the primitive ectoderm marker Fgf5 [35]. The peak of the early neural marker Pax6 [36] at day 5 indicated the occurrence of neuronal induction, anticipating the expression of the MN-specific markers Hb9 [37] and ChAT [38] at later stages. The timing of marker expression in FUS^HOMO^ was the same as in FUS^WT^ and FUS^KO^ lines (Online resource 3: Fig. S1a and S1c), indicating that neither the absence nor the mutation of FUS affects the differentiation potential of mESCs, in agreement with previous data [21]. Progression along MN differentiation was also checked by monitoring the expression of a GFP transgene [33], driven by the MN-specific promoter Hb9 (Fig. 1a). After 6 days of differentiation, the GFP expression allowed the selection of MNs [GFP(+) cells], corresponding to about 40% of the mixed neural cell population obtained upon embryoid body (EB) dissociation (Fig. 1c). GFP(+) cells were characterized by robust expression of genes known to play important functions in MN development (Islet-1) [39] as well as cell identity acquisition (Hb9) and function (ChAT) (Fig. 1d). Conversely, the complementary GFP(−) cell population was enriched for Pax6 and Olig2 transcripts, highly expressed in neural and MN precursors, respectively, demonstrating that the GFP(−) fraction was largely composed of neural progenitors. Similar results were obtained when we analyzed GFP(+) and GFP(−) cells derived from either FUS^WT^ (Online resource 3: Fig. S1b) or FUS^KO^ (Online resource 3: Fig. S1d) lines, again indicating that in vitro differentiation of spinal MNs was not affected by alteration of FUS abundance or activity.

**Fig. 2:**
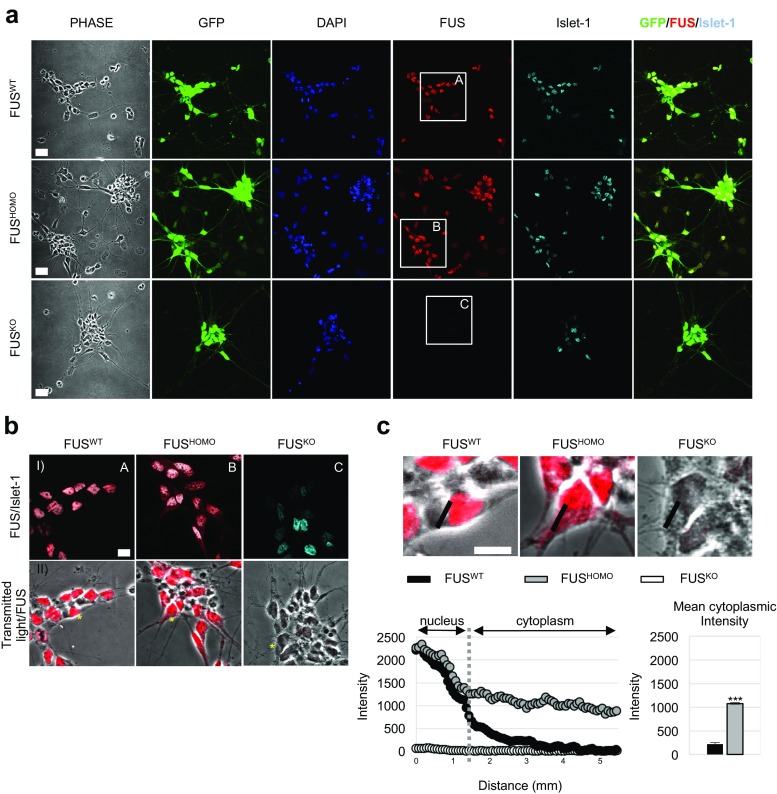
FUS protein localization in mESC-derived MNs: **a** Multiple immunostaining analysis of FUS (red) and the MN marker Islet-1 (cyan) in FUS^WT^, FUS^HOMO^, and FUS^KO^ mixed cell populations cultured for 2 days after EB dissociation and replating. The higher GFP signal is mainly located in Islet1(+) cells. No significant FUS signal was found in FUS^KO^ cells. Nuclear staining with DAPI (4′,6-diamidino-2-phenylindole) is reported in blue. Scale bar: 20 μm. **b** Magnification of square inserts (A, B, C) reported in **a** showing FUS and Islet-1 double immunofluorescence (row I) or FUS staining combined with transmitted light (row II). **c** Representative line scan analysis of the FUS signal intensity in MNs indicated by asterisks in B (row II). A fluorescent intensity value along the black line drawn across the nucleus and cytoplasm (upper panel) was plotted versus distance (bottom chart). Note the shift in cytoplasmic intensity profile in FUS^HOMO^ (gray dots) with respect to FUS^WT^ (black dots) and FUS^KO^ (white dots). The histogram on the left represents the mean (+/− SEM) of cytoplasmic intensity between FUS^WT^ and FUS^HOMO^ MNs. Scale bar: 10 μm. Results (+/− SEM) are expressed in arbitrary units

FUS mRNA expression and protein localization were then analyzed in GFP(+) MNs derived from FUS^WT^, FUS^HOMO^, and FUS^KO^. The results indicate that, while the mRNA levels are unaffected (Online resource 3: Fig. S2), the subcellular localization of the protein changes with an approximately fivefold increase in the cytoplasmic compartment (Fig. 2a, b) compared to FUS^WT^ (Fig. 2c).

### Transcriptome Analysis of FUS-Depleted or FUS-Mutant MNs

To investigate the effects of FUS mutation on the MN transcriptome, we analyzed global gene expression through Ribo-Zero NGS, in FUS^HOMO^ MNs differentiated from three independent mESC lines. From a technical point of view (sequencing depth, read length and pairing, sequencing center of origin, mapping statistics), RNA-Seq data were comparable with those previously produced in the lab for the FUS^WT^ and FUS^KO^ genetic backgrounds [25]. Almost 90% of the reads were successfully mapped to the mouse genome (Online resource 4: Table 2), with the majority aligned to unique locations. Mitochondrial RNAs, rRNAs, tRNAs, snRNAs, snoRNAs, miRNAs, and other non-coding species shorter than 200 nt were excluded from further analysis. We found that the transcriptome of FUS^WT^ MNs (Online resource 3: Fig. S3) consists of 36,725 expressed RNAs (FPKM > 0.1) corresponding to 14,473 unique gene loci, 12,895 of which encode for proteins.

Differential gene expression analysis was performed to identify mRNAs whose levels changed in FUS^KO^ and FUS^HOMO^, compared to FUS^WT^. We distinguished three categories of differentially expressed genes, accounting for the effect of gain or loss of function upon FUS mutation. The first group includes the genes altered exclusively in FUS^KO^ and therefore directly due to a loss-of-function mechanism. The second comprises those altered both in FUS^KO^ and FUS^HOMO^; these could represent a class of transcripts more sensitive to the nuclear levels of FUS. The third group is composed of transcripts affected only in FUS^HOMO^ which are likely to be due to a gain of function of the mutant FUS, either in the nucleus or in the cytoplasm.

**Fig. 3:**
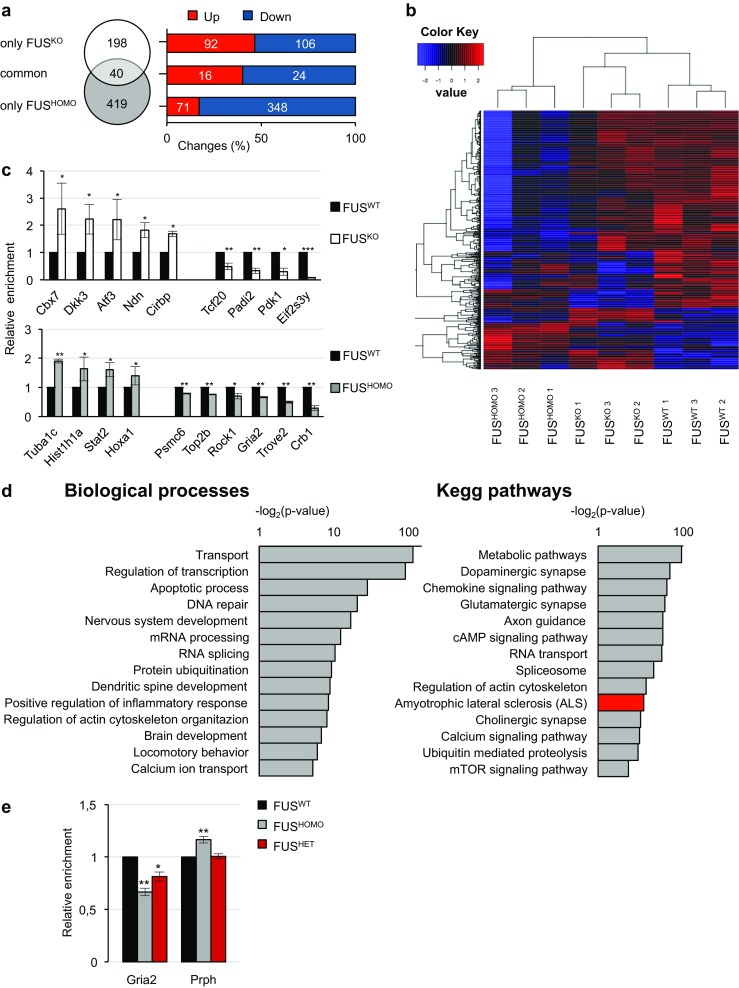
Differential gene expression analysis in FUS^KO^ and FUS^HOMO^ MNs. **a** Left: Venn diagram showing the number of genes misregulated exclusively in FUS^KO^ (white circle) or in FUS^HOMO^ (gray circle) MNs. Forty genes are commonly deregulated (light gray area). Right: distribution of differentially expressed genes according to deregulation tendency (red: upregulation; blue: downregulation). **b** Heat map shows the relative expression levels of differentially expressed genes, along with the hierarchical clustering of genes and samples (three biological replicates). Expression levels used in the heatmap were calculated by mean-centering the log2-transformed FPKM values of genes. The heatmap represents only those genes having FPKM > 1 in at least three samples. **c** qRT-PCR analysis of selected mRNAs differentially expressed in FUS^KO^ (upper histogram, white bars) or FUS^HOMO^ (lower histogram, gray bars) compared to FUS^WT^ (black bars), set as 1. Results (means +/− SEM) from three biological replicates are expressed in arbitrary units and are normalized to the mean value of Atp5o mRNA. *p < 0.05, **p < 0.01, ***p < 0.001 two-tailed Student’s *t* test. **d** Representative functional categories of genes differentially expressed between FUS^HOMO^ and FUS^WT^ according to Gene Ontology analysis. Biological processes (left diagram) or KEGG pathways (right diagram) are shown. The ALS gene category is highlighted in red. **e** qRT-PCR analysis of Gria2 and Prph in FUS^HOMO^ (gray bars) and FUS^HET^ (red bars) compared to FUS^WT^ (black bars), set as 1. Results (means +/− SEM) from three biological replicates are expressed in arbitrary units and are normalized to the mean value of Atp5o mRNA. *p < 0.05, **p < 0.01, two-tailed Student’s *t* test

When compared to FUS^WT^, 238 protein-coding genes proved to be differentially expressed in FUS^KO^ (108 genes were upregulated and 130 were downregulated); of these, 40 were significantly modulated both in FUS^KO^ and FUS^HOMO^, whereas 419 were deregulated exclusively in FUS^HOMO^ (348 genes were downregulated and 71 were upregulated) (Fig. 3a and Online resource 5: Table 3). The heatmap of differentially expressed genes is reported in Fig. 3b. Based on expression levels (FPKM > 1), fold change [log2(fold change) > 0.5], and/or biological significance, hits from the two main lists of differentially expressed genes, i.e., those specifically altered between FUS^HOMO^ and FUS^WT^ and those modulated only upon FUS depletion, were picked out for RNA-Seq validation. Representative candidate genes matching these criteria were analyzed by qRT-PCR. Data shown in Fig. 3c confirm the RNA-Seq data for all of them.

### The Gria2 Subunit of the AMPA Receptor Is Downregulated in FUS-Mutant MNs

By using the DAVID Functional Annotation Tool [40], we performed Gene Ontology (GO) term enrichment analyses of the genes specifically altered in FUS^HOMO^ or in FUS^KO^ conditions (Online resource 6 : Table 4). Selected functional categories (reported in the left panel of Fig. 3d for FUS^HOMO^ and in Online resource 3: Fig. S4 for FUS^KO^) include a large number of genes participating in general biological processes such as transcription, splicing, and cell physiology; moreover, a conspicuous number of genes regulating neuronal activity, development, and function were also found. KEGG pathway enrichment analysis performed on genes differentially expressed in FUS^HOMO^ (Fig. 3d, right panel) identified a molecular pathway named “amyotrophic lateral sclerosis,” composed of three hits, whose deregulation was already associated with ALS: Cyct (cytochrome C) [41], Prph (peripherin) [42–44], and Gria2 (glutamate ionotropic receptor α‐amino‐3‐hydroxy‐5‐methyl-4-isoxazole propionic acid (AMPA) type subunit 2) [45, 46].

As ALS-causative FUS mutations are autosomal dominant, we analyzed a related ESC line, derived from a heterozygous mouse for the FUS-P517L mutation (N. Shneider, unpublished) matching the patients’ genotype. Since Cyct was expressed at very low levels (FPKM < 1), we focused on Gria2 and Prph (FPKM > 20) to test whether their expression was altered in FUS^HET^ MNs. Figure 3e shows that Gria2 mRNA was downregulated in FUS^HET^ MNs at an intermediate level between FUS^WT^ and FUS^HOMO^ indicating a dose effect of the FUS mutation on Gria2 expression. Conversely, Prph which was slightly upregulated in FUS^HOMO^ did not show any change in FUS^HET^ MNs. Interestingly, reduced Gria2 levels specifically characterize post-mitotic FUS^HET^ and FUS^HOMO^ GFP(+) MNs, while no effect is observed in the GFP(−) population (Online resource 3: Fig. S5).

### mRNA/miRNA Cross-Analysis in FUS-Depleted and FUS-Mutant MNs

Since miRNAs have been shown to participate in MN metabolism [47–49] and to be affected by FUS mutations [50], we used NGS to analyze the global miRNA expression profiles in mESC-derived FUS^WT^, FUS^HOMO^, and FUS^KO^ MNs. Reads produced in this experiment were mapped to a database of known and predicted miRNA isoforms (mapping statistics are reported in Online resource 7: Table 5). Five hundred sixty-five miRNAs had at least one read per million mapped to them in FUS^WT^ MN samples.

**Fig. 4:**
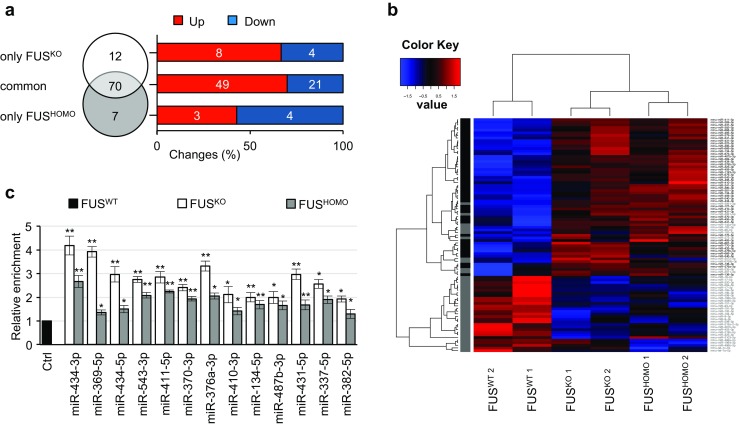
Analysis of microRNA differential expression in FUS^KO^ and FUS^HOMO^ MNs. **a** Left: Venn diagram showing the number of microRNAs misregulated exclusively in FUS^KO^ (white circle) or in FUS^HOMO^ (gray circle) MNs. Seventy genes are commonly deregulated (light gray area). Right: distribution of differentially expressed microRNAs according to deregulation tendency (red: upregulation; blue: downregulation). **b** Heatmap showing the relative expression levels of differentially expressed microRNAs, along with the hierarchical clustering of miRNAs and samples. Expression levels used in the heatmap were calculated by mean-centering the log2-transformed full quantile-normalized read counts of microRNAs. miRNAs belonging to the *Dlk1-Dio3* cluster are highlighted in bold. **c** qRT-PCR analysis of selected microRNAs differentially expressed in FUS^KO^ (white bars) and FUS^HOMO^ (gray bars) compared to FUS^WT^ (black bars), set as 1. Results (means +/− SEM) from three biological replicates are expressed in arbitrary units and are normalized to the mean value of U6 snRNA. *p < 0.05, **p < 0.01, two-tailed Student’s *t* test

Through differential expression analysis, we identified seven miRNAs that were exclusively deregulated in FUS^HOMO^ compared to FUS^WT^ MNs (three upregulated and four downregulated) and 12 miRNAs that displayed an altered expression only in FUS^KO^ (eight upregulated and four downregulated). However, a large number of miRNAs (70) were concordantly deregulated both in FUS^HOMO^ and FUS^KO^ MNs, 70% of which resulted upregulated in both conditions (Fig. 4a and Online resource 8: Table 6). For this class of RNAs, the similar expression patterns in FUS^HOMO^ and FUS^KO^ MNs suggested a predominant loss-of-function effect [50]. Analysis of the genomic distribution of the miRNAs upregulated both in FUS^HOMO^ and FUS^KO^ indicated that more than 85% of them localize to a single genetic *locus*, namely, *Dlk1-Dio3*, on the distal portion of chromosome 12. Furthermore, all of the miRNAs encoded from this *locus* were upregulated in FUS^HOMO^ and FUS^KO^ MNs. The heatmap of differentially expressed genes is reported in Fig. 4b. qRT-PCR analysis of 13 miRNAs belonging to this subgroup validated the RNA-Seq data (Fig. 4c).

**Fig. 5:**
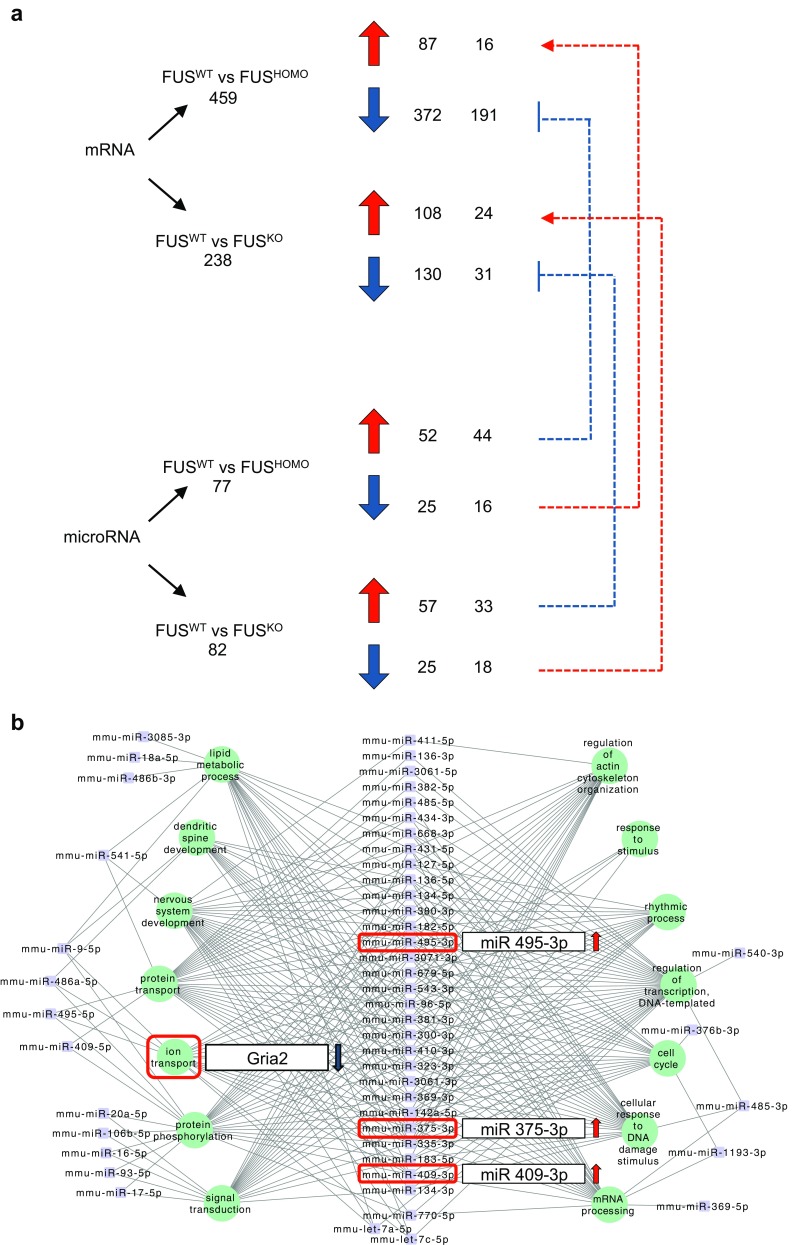
Differentially expressed mRNA and miRNA cross-analysis. **a** Diagram showing, for each sample, the numbers of upregulated (red arrows) and downregulated (blue arrows) mRNAs and microRNAs, with opposing differential expression patterns. Blue dashed lines and red dashed arrows lines indicate negative and positive interactions between microRNAs and mRNAs, respectively. **b** Networking between upregulated microRNAs (purple dots) and downregulated predicted targets mRNAs (clustered in functional categories and represented as green dots) in FUS^HOMO^ MNs. Ion transport category, Gria2 gene, and microRNAs (miR-409-3p, miR-495-3p, and miR-375-3p) are highlighted

We then crossed the data from the FUS^HOMO^ long and small RNA sequencing to identify possible candidates converging on common pathways. Figure 5a shows the results of the analysis of miRNAs and their putative target mRNAs (identified through DIANA-microT software [30], Online resource 9: Table 7) exhibiting anti-correlated expression. The most abundant gene subgroup included 191 transcripts, which were downregulated in FUS^HOMO^ condition along with upregulation of putative effector miRNAs. Such a large miRNA-mRNA regulative cross talk was not observable in FUS^KO^. As shown in Online resource 3: Fig. S6, the classes of genes deregulated only in FUS^KO^ or in both conditions show a significantly lower percentage of mRNAs putatively targeted by miRNAs with anti-correlated expression compared to the genes altered in FUS^HOMO^ (*p* values for chi-squared test = 1.666e−08 and 0.0004114, respectively). This fact suggests that the effect of deregulated miRNAs on their targets is poorly contributed by FUS loss of function.

Genes downregulated in FUS^HOMO^ and targeted by upregulated miRNAs were organized into functional categories by GO analysis and combined together with their putative interacting miRNAs into functional circuitries through Cytoscape software [51] (Fig. 5b). Of note, among functional categories involved in ALS/neurodegeneration such as lipid metabolism process [52], regulation of actin cytoskeleton organization [53], protein transport [54], and DNA repair [55], we identified a cluster of genes named “Ion Transport.” It included Gria2, which was predicted to be targeted by miR-409-3p, miR-495-3p, and miR-375-3p at multiple sites. Consistent with their involvement in a common circuitry, Gria2 mRNA was downregulated in FUS^HOMO^ conditions where the three miRNAs were upregulated.

### Gria2 Is Targeted by miR-409-3p and miR-495-3p

**Fig. 6:**
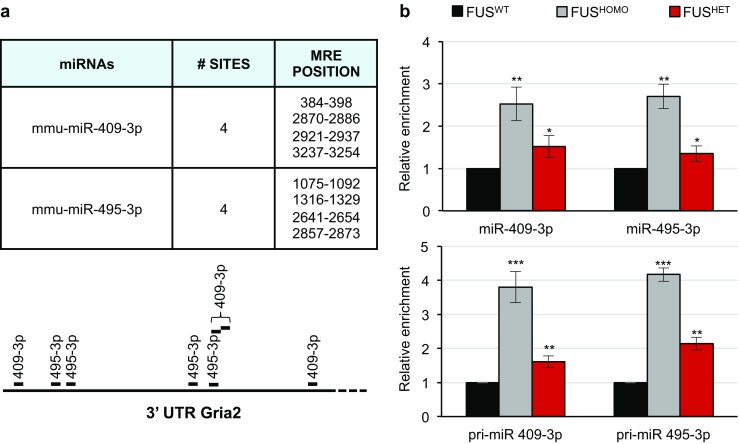
Analysis of miR-409-3p and miR-495-3p expression. **a** Numbers and positions (referred to TSS of Gria2 mRNA) of miR-409-3p and miR-495-3p predicted MREs on Gria2 3′-UTR. Schematic localizations of the eight MREs on the Gria2 3′-UTR are represented below. **b** Upper panel: qRT-PCR analysis of miR-409-3p and miR-495-3p expression in FUS^HOMO^ (gray bars) and FUS^HET^ (red bars) MNs, compared to FUS^WT^ (black bars), set as 1. Results (means +/− SEM) from three biological replicates are expressed in arbitrary units and are normalized to the mean value of U6 snRNA. *p < 0.05, **p < 0.01, two-tailed Student’s *t* test. Lower panel: qRT-PCR analysis of pri-miR-409-3p and pri-miR-495-3p expression in FUS^HOMO^ (gray bars) and FUS^HET^ (red bars) MNs, compared to FUS^WT^ (black bars), set as 1. Results (means +/− SEM) from three biological replicates are expressed in arbitrary units and are normalized to the mean value of Atp5o mRNA. *p < 0.05, **p < 0.01, ***p < 0.001 two-tailed Student’s *t* test

**Fig. 7:**
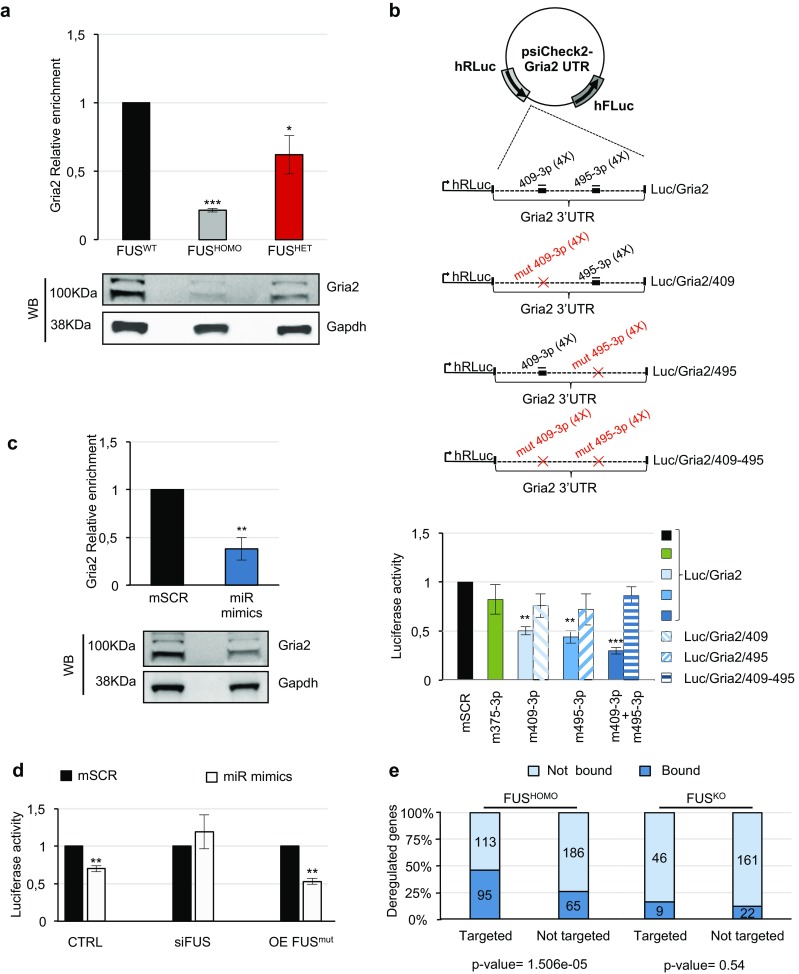
Analysis of interactions between miR-409-3p and miR-495-3p and Gria2. **a** Western blot analysis of Gria2 protein in FUS^HOMO^ and FUS^HET^ compared to FUS^WT^ MNs, set as 1. Protein-level densitometric analysis is reported above. Results (means +/− SEM) from three biological replicates are expressed in arbitrary units and are normalized to the mean value of the GAPDH protein. *p < 0.05, ***p < 0.001, two-tailed Student’s *t* test. **b** Luciferase assay in N2A cells. Upper panel: representation of luciferase/Gria2 gene 3′-UTR reporter constructs. MREs are indicated as thick lines, microRNAs as thin lines. For each microRNA, number of MREs in the construct is reported in brackets. Red crosses indicate mutations in derivative constructs. Lower panel: activity of Renilla luciferase expressed from Luc/Gria2 constructs, in the presence of scrambled (mSCR) or specific microRNA mimics (m409-3p, m495-3p, and m375-3p) transfected as single molecules or in combination, as indicated below each bar. Full bars or striped bars indicate luciferase activity from WT or MRE-mutated Luc/Gria2 constructs, respectively (as specified aside). Renilla luciferase activity (means +/− SEM) from three biological replicates is expressed in arbitrary units, normalized over Firefly luciferase activity as internal control, and referred to SCR sample, set as 1. **p < 0.01, ***p < 0.001, two-tailed Student’s *t* test. **c** microRNAs regulate Gria2 gene expression. Western blot analysis of Gria2 protein in dissociated day 6 EBs transfected with control mimic (mSCR, set as 1) or with a combination of specific mimics for miR-409-3p and miR-495-3p (miR mimics). Results (means +/− SEM) from three biological replicates are expressed in arbitrary units and are normalized to the mean value of the GAPDH protein. **p < 0.01, two-tailed Student’s *t* test. **d** Luciferase assay upon FUS modulation. Activity of Renilla luciferase expressed from the Luc/Gria2 construct, in the presence of scrambled (black bars) or specific microRNA mimics (m409-3p and m495-3p) transfected in combination (light gray) in uncommitted N2A cells (CTRL) or cells overexpressing FUS P525L (OE FUS^mut^) or cells interfered for FUS (siFUS). Renilla luciferase activity (means +/− SEM from three biological replicates) is expressed in arbitrary units, normalized over Firefly luciferase activity as internal control, and referred to scrambled sample, set as 1. **p < 0.01 two-tailed Student’s *t* test. **e** Cross-analysis between differentially expressed mRNAs targeted by miRNAs in FUS^HOMO^ and FUS^KO^ condition and FUS CLIP-Seq data. The bar plot shows that, according to a reanalysis of the CLIP-Seq dataset [15], mRNAs deregulated in FUS^HOMO^ condition and putatively targeted by anti-correlated miRNAs are also enriched for FUS-binding sites at the 3′-UTR level, compared to non-targeted deregulated genes

According to different software (DIANA-microT-CDS and TargetScan [56]), only the miR-409-3p and miR-495-3p-responsive elements (MREs) proved to be fairly well conserved in the mouse and human Gria2 3′-UTR; instead, miR-375-3p was not predicted by both software, and it displayed suboptimal and mainly non-conserved MREs. Therefore, we focused our analysis on miR-409-3p and miR-495-3p (Fig. 6a). Both of these are transcribed from the abovementioned *Dlk1-Dio3 locus*, in the highly conserved and brain-expressed cluster miR379-410. As shown in the upper panel of Fig. 6b, upregulation of the two miRNAs was validated by qRT-PCR in FUS^HOMO^ and FUS^HET^ MNs. In line with what was observed for the mature species, we found that also miR-409-3p and miR-495-3p primary transcripts increased in FUS^HOMO^ MNs, whereas an intermediate level of enrichment was detected in FUS^HET^ condition (Fig. 6b, lower panel). In parallel, we checked the Gria2 protein levels in the same samples and observed a significant downregulation of the Gria2 protein (Fig. 7a): compared to FUS^WT^, a larger decrease was observed in FUS^HOMO^ (about 60% reduction), whereas an intermediate level was detected in FUS^HET^ (40% decrease). By normalizing these values with the amount of the Gria2 mRNA, we could define that FUS mutation had an effect at both mRNA and protein levels (compare Fig. 7a with Fig. 3e). These results suggested a specific direct correlation between Gria2 and miR-409-3p and miR-495-3p. To verify this interplay, we cloned the 3′-UTR of Gria2 downstream of a luciferase reporter (schematized in Fig. 7b) and ectopically expressed this construct in murine neuronal N2A cells along with miR-409-3p and miR-495-3p mimics. As shown in the histogram of Fig. 7b, cells overexpressing either of the two miRNAs along with the Luc/Gria2 reporter exhibited an approximately 50% decrease in luciferase activity, compared to the scrambled control. Co-transfection of the two miRNAs further repressed luciferase activity, while no effect was observed with miR-375-3p. Luciferase activity was partially recovered in cells transfected with miR-409-3p or miR-495-3p mimics and the respective mutant sensors Luc/Gria2/409 or Luc/Gria2/495 (each carrying selective miR-409-3p or miR-495-3p MRE mutations impairing miRNA binding); an almost full rescue was obtained in the double mutant. These results confirm the specificity of the miRNA/Gria2 interaction. The overall results confirmed that miR-409-3p and miR-495-3p specifically and synergistically control Gria2 mRNA translation/stability by targeting its 3′-UTR. As a further demonstration of this regulatory network, the overexpression of miR-409-3p and miR-495-3p in FUS^WT^ ESC-derived EBs produced the downregulation of the Gria2 endogenous protein (Fig. 7c).

However, we noticed that the Gria2 mRNA levels were lower in homozygous than in knock-out condition even though the targeting miRNAs were upregulated at similar levels. Since FUS is also known to bind the 3’UTR of mRNAs, we wondered whether FUS could cooperate with miRNAs by binding the 3′-UTR of Gria2, thus leading to its specific decrease in FUS^HOMO^ MNs. Analysis of available CLIP-Seq data in wild-type FUS mouse brain indeed confirmed the binding of FUS to the 3′-UTR of Gria2 [15]. Therefore, we repeated the luciferase assays upon modulation of FUS expression in N2A cells. Compared to control, FUS silencing was able to counteract the miRNA-dependent downregulation of luciferase, whereas the overexpression of the FUS mutant allele P525L enhanced the miRNA repression activity on the Gria2 reporter (Fig. 7d). These data indicated that FUS is required for the control exerted by miR-409-3p and miR-495-3p on Gria2 mRNA stability and translation and explain why in the KO condition no effects on the levels of Gria2 are observed.

**Fig. 8:**
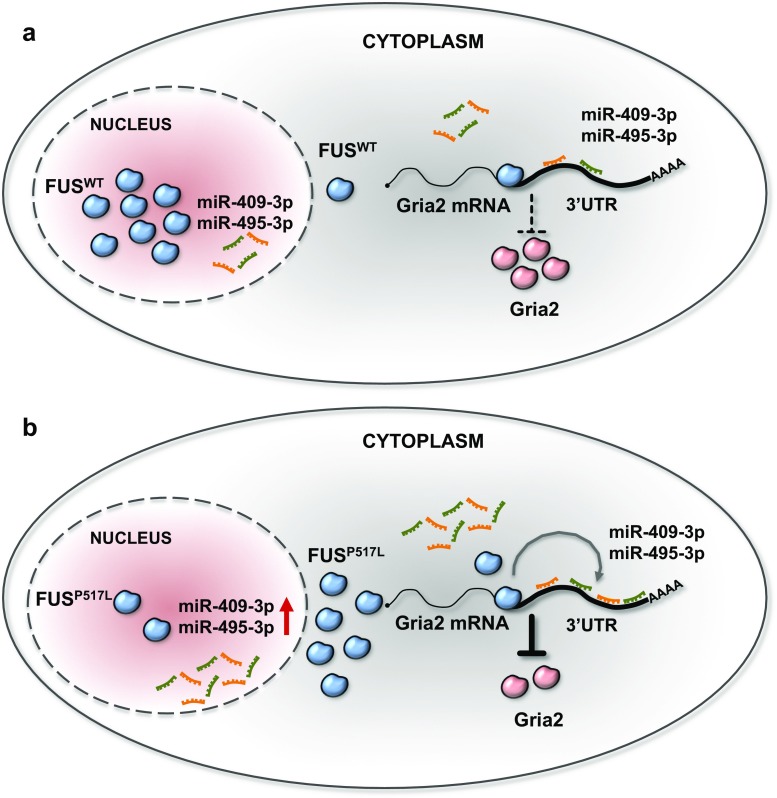
FUS-dependent post-transcriptional regulation of Gria2. **a** Gria2 is under the control of miR-409-3p and miR-495-3p. **b** Upregulation of miR-409-3p and miR-495-3p (dependent on nuclear FUS depletion) synergizes with FUS-P517L cytoplasmic delocalization to trigger a negative regulatory loop repressing Gria2 expression

To test whether this is a more general phenomenon, we reanalyzed available CLIP-Seq data in wild-type FUS mouse brain and we found that, in the FUS^HOMO^ condition alone, deregulated mRNAs, putatively targeted by miRNAs with an anti-correlated expression, are preferentially bound by FUS in their 3′-UTR, compared to those deregulated mRNAs which are not targeted (*p* value for chi-squared test = 1.506e−05 and 0.5416 for FUS^HOMO^ and FUS^KO^ conditions, respectively) (Fig. 7e). This strong association between FUS and mRNAs deregulated in FUS^HOMO^ condition, which are targeted by differentially expressed microRNAs, supports our hypothesis that FUS mediates microRNA activity (Fig. 8).

In conclusion, our data underline the existence of a complex network of regulatory interactions where FUS controls not only the biogenesis of miRNAs but also their activity on target mRNAs. In the case of the Gria2 circuitry, the decrease in the nuclear levels of FUS-P517L leads to miRNA upregulation while the concomitant increase in the mutant protein in the cytoplasm synergizes with miRNA activity, therefore reinforcing the repressive loop on Gria2.

## Discussion

The well-established role of FUS in RNA metabolism and in MN degeneration, together with the observed cytoplasmic mislocalization of mutant FUS proteins, raises the important question of whether any crucial RNA-mediated regulatory circuitry contributes to the pathogenesis of ALS. In this study, we have analyzed the small and long RNA transcriptomes of mouse MNs carrying a knock-in allele FUS-P517L (equivalent to the ALS-associated human P525L mutation) to identify specific regulatory networks based on the cross talks between miRNAs and the corresponding target mRNAs.

As far as the mRNA transcriptome was concerned, we found that less than 10% of the mRNAs deregulated in the FUS P517L homozygous MNs were also perturbed in FUS^KO^ MNs, and therefore linked to a loss-of-function mechanism. At variance, the majority were not shared with FUS^KO^ indicating a gain-of-function effect in the nucleus or in the cytoplasm [10, 14, 57, 58]. Since previous reports have described the ability of FUS to bind transcripts [13, 16], it is plausible that at least a fraction of deregulated mRNAs respond to altered FUS association. However, several studies also suggest the relevance of FUS activity in miRNA biogenesis and in MN function [50, 59]; thus, it is possible to envisage that FUS mutations may also affect mRNA expression indirectly, via deregulation of miRNA levels. Small-RNA sequencing analysis highlighted that the majority of miRNAs were similarly deregulated upon FUS mutation and knockout, suggesting a mechanism of loss of function for the biogenesis of these transcripts.

Bioinformatic predictive analysis, performed on the entire repertoire of mRNA transcripts putatively targeted by multiple differentially expressed miRNAs and belonging to at least two different families, revealed clustering of well-defined gene categories (Online resource 3: Fig. S7). Among these, there is a cluster (amyotrophic lateral sclerosis) composed of 12 genes linked to ALS through different pathogenic mechanisms such as protein misfolding/ER stress (Derlin1), MAPK signaling (Ask1, p38), mitochondrial pathway of cell death (Bcl-2, Bcl-2l1, Apaf1), and Ca^2+^ dysregulation (calcineurin). Even though these mRNA targets did not appear deregulated, it cannot be excluded that the control occurs uniquely at the translational level, without affecting the stability of the mRNAs. Instead, the comparative analysis of small- and long-RNA variations occurring in FUS^HOMO^ MNs identified almost 50% of the FUS^HOMO^ mRNAs inversely correlating with their putative regulatory miRNAs. The majority of these mRNAs proved to be downregulated with the targeting miRNAs being upregulated; GO analysis revealed that they participate into gene pathways involved in neurodegeneration (cytoskeleton organization, DNA repair, protein or ion transport, lipid metabolism). One interesting case was represented by the repression of the Gria2 AMPA subunit mRNA, which paralleled the upregulation of miR-409-3p and miR-495-3p. Further tests allowed the validation of Gria2 as a bona fide target of both miRNAs and showed that the protein levels of Gria2 were indeed altered as a consequence of the miRNA upregulation. Notably, the same reciprocal modulation and altered Gria2 levels were found in MNs carrying the FUS-P517L mutation in heterozygosity, which perfectly matches the genetic background of the human condition, suggesting that this circuitry could indeed be involved in the ALS pathology.

miR-409-3p and miR-495-3p are both transcribed from the *Dlk1-Dio3* imprinted *locus*, which spans 800 kb in the long arm of mouse chromosome 12. It is conserved in humans [60, 61] and, together with several non-coding RNA genes, contains more than 50 miRNAs. Gene regulation of *Dlk1-Dio3* is unclear: both transcriptional and post-transcriptional events have been proposed to drive whole-locus or single-gene expression [62]. Our RNA-Seq data indicate that all the miRNAs of the *Dlk1-Dio3* cluster are similarly upregulated in FUS^KO^ or in FUS^HOMO^ and wild-type FUS CLIP-Seq reanalysis did not show any specific enrichment for FUS binding in the pri-miRNA regions harboring miR-409-3p and miR-495-3p. These data favor the hypothesis of a transcriptional effect of FUS. Consistently, pri-miR-409-3p and pri-miR-495-3p are detected at higher levels in FUS^HOMO^ conditions. From the available data, we cannot conclude whether these effects are due to a direct or indirect effect of FUS.

Notably, deregulation of the *Dlk1-Dio3 locus* has been associated with a number of pathologies [63–68]. In particular, miR-409-3p and miR495-3p belong to a brain-specific subcluster, named miR379-410. This participates in neuronal development, maturation, and function, and it has been proposed that its deregulation contributes to the onset of several neurodevelopmental disorders, such as epilepsy, schizophrenia, and autism, as well as brain tumors [23]. However, to date, no clear activity has been selectively attributed to the two miRNAs in neuron physiopathology. Our results indicate a novel regulatory function for these miRNAs that impinges on Gria2, a protein involved in ALS and implicated in MN-specific sensitivity to the pathology. In fact, several studies have demonstrated that reduction of either editing [69–71] or expression [69, 72, 73] of Gria2 is linked to MN degeneration in ALS through disturbance of Ca^2+^ homeostasis which triggers a cascade of damaging “excitotoxic” events.

We also show that the activity of miR-409-3p and miR495-3p is promoted by FUS itself, which is known to bind the Gria2 3′-UTR. This is the first time that such cooperation between FUS and miRNA activity has been identified. We propose that this is likely to be a more general feature since CLIP-Seq data in mouse brain have indicated that deregulated mRNAs, putatively targeted by miRNAs with an anti-correlated expression, are preferentially bound by FUS in their 3′-UTR compared to those which are not targeted. No FUS CLIP-Seq data are currently available for murine mutation or human FUS mutations, in neuronal cells. However, previous studies indicate that mutations in the C-terminal domain of FUS did not alter its binding capability and specificity [50, 74].

In conclusion, our study characterizes for the first time the small- and long-RNA transcriptomes of MNs which carry one of the most severe types of FUS mutation and identifies miRNA/mRNA regulatory circuits, which can be directly linked to the pathology.

Moreover, in the case of Gria2, a subunit of the glutamate AMPA receptor already linked to MN physiology and ALS pathogenesis, we have identified a specific negative regulatory loop (i) mediated by the upregulation of miR-409-3p and miR-495-3p and (ii) reinforced by the altered nucleus/cytoplasmic partitioning of FUS-P517L. In fact, the increase in expression of the two miRNAs due to the decrease of the nuclear levels of mutant FUS synergizes with the higher levels of the protein in the cytoplasm which strengthens miRNA-repressing activity (Fig. 6).

## Electronic supplementary material

ESM 1 (XLSX 40 kb)

ESM 2 (DOCX 37 kb)

ESM 3 (PDF 140 kb)

ESM 4 (XLSX 45 kb)

ESM 5 (XLSX 81 kb)

ESM 6 (XLSX 159 kb)

ESM 7 (XLSX 41 kb)

ESM 8 (XLSX 19 kb)

ESM 9 (XLSX 35 kb)

ESM 10 (DOCX 21 kb)
