# Supplementary material for: A Regulatory Circuitry Between Gria2, miR-409, and miR-495 Is Affected by ALS FUS Mutation in ESC-Derived Motor Neurons
Source: Mol Neurobiol. 2018 Feb 12;55(10):7635–51. doi: 10.1007/s12035-018-0884-4 (PMC6132778; doi:10.1007/s12035-018-0884-4)
Supplement: Supplementary file 2 — (DOCX 37 kb) [file 12035_2018_884_MOESM2_ESM.docx]

**Supplementary Methods**

**Cell cultures and treatments.**

Generation of embryoid bodies (EBs) was achieved by culturing mESCs in ADNFK medium (1:1 Advanced DMEM/F12:Neurobasal medium, 10% Knock Out Serum Replacement (Gibco, 10828028), 1% GlutaMAX, 1% 2-mercaptoethanol, 1% Pen/Strep). On day 2, ADNFK medium was complemented with 2% B27 Supplement (Gibco, 17504-044), 1μM RA (Sigma Aldrich, R2625) and 0.5 μM SAG (Merck Millipore, 566660). EBs were expanded and then disrupted by Papain dissociation system (Worthington Biochemical Corporation) following the manufacturer’s instructions.

**Isolation of motoneurons by FACS**

To reduce sorting-associated-stress, MNs were isolated in “gentle conditions” using a ceramic nozzle of size 100 μm, a low sheath pressure of 19.84 pound-force per square inch (psi) that keep the sample pressure at 18.96 psi and an acquisition rate of 4000 events/s. Analysis was performed using FlowJo software (Tree Star): cells first gated based on forward and side scatter area (FSC-A and SSC-A) plot were then detected in the green fluorescence channel for GFP expression (530/30 nm filter) and collected as negative [GFP(-)] and positive cells [GFP(+)].

Upon isolation, a small aliquot of each collected sample (5 and/or 15 ml polystyrene or polypropylene tubes according to the different use) was evaluated for purity at the same sorter resulting in an enrichment >98-99% for each sample.

**RNA-Seq and bioinformatics analysis**

RNA-Seq reads were initially trimmed using the Trimmomatic software [1] to remove adapter sequences and poor quality bases; the minimum read length after trimming was set to 30. Bowtie 2 was used to align reads to a sequence database composed of rRNAs, tRNAs, snRNAs, snoRNAs and other non-coding species which resulted to be overrepresented according to FastQC software available online at: http://www.bioinformatics.babraham.ac.uk/projects/fastqc; reads mapping to these sequences were filtered out. In order to calculate the distribution of the inner distance between mate pairs, reads were aligned to a non-redundant set of mRNA sequences derived from Ensembl 77 gene annotation [2] using BWA software [3]. Mean and variance of inner distance distribution were estimated from aligned read pairs whose inner distance was within interval [Q1-2(Q3-Q1), Q3+2(Q3-Q1)] (Q1=first quartile, Q3=third quartile). TopHat2 [4] was employed to align reads to GRCm38 mouse genome and Ensembl transcriptome using parameters -i 50 -r 36 --mate-std-dev 73 --library-type fr-firststrand. Samtools [5] was used to filter off reads mapping to mitochondrial genome. Gene-level and isoform-level expression estimation, as well as differential expression analysis, were performed using Cuffdiff 2 software [6]. Each Ensembl gene was assigned a FPKM value equal to the sum of the FPKM values of its transcripts; this was done to disambiguate cases in which Cuffdiff merges two or more Ensembl genes because of their proximity.

To select differentially expressed genes, we first used a p-value cutoff for the gene-level differential expression equal to 0.05. Then, among the genes whose q-value was < 0.1, we selected only those whose maximum FPKM value in one condition was lower or higher (depending on the fold-change direction) than the average FPKM in the other condition; genes having q-value > 0.1 (but still with p-value < 0.05) were selected only if the maximum FPKM value in one condition was lower than the minimum FPKM in the other condition multiplied by 0.9. Genes having a transcript with a transcript-level differential expression q-value < 0.1 and whose maximum FPKM value in one condition was lower or higher (depending on the fold-change direction) than the average FPKM in the other condition were also added to the list of differentially expressed genes. Gene expression heatmaps were generated using Heatmap3 R package [7].

**Small RNA-Seq analysis**

Adapter sequences were removed using Trimmomatic software, using a simple clip threshold equal to 8. After trimming, reads whose length was less thab 18 bp were discarded. Bowtie with --best and -v 0 switches was then used to align remaining reads to a sequence database composed of canonical microRNAs and their putative isoforms; this database, derived from mirBase 21, is available at http://cru.genomics.iit.it/Isomirage/. To estimate the expression of each mature microRNA, we used the number of reads mapping to its template isoforms calculated using the IsomiRage software [8]. Poorly expressed miRNAs, defined as those not achieving one count per million in at least two samples, were filtered out. Full quantile normalization was then applied to raw read counts using the EDASeq R package [9]; normalized read counts were supplied to EdgeR [10] for differential expression analysis. This analysis yielded 44 and 55 miRNAs, respectively deregulated in FUS mutant and FUS KO MNs (FDR < 0.1). A large number of miRNAs (27 RNAs, accounting for 37.5% of all altered microRNAs) were differentially expressed in both conditions, compared to FUS^WT^. A manual inspection of miRNA expression values suggested that those just below the FDR threshold seem to be effectively deregulated; since we did not want to lose information on miRNAs deregulated in both conditions, which represent a high proportion of all the deregulated miRNAs and thus appear to be very important, we decided to extend the lists of differentially expressed miRNAs by including those with same fold-change direction in both conditions, using a less stringent p-value cutoff (unadjusted p-value < 0.05).

**CLIP-Seq data reanalysis**

We identified FUS CLIP-Seq peaks for each of the three biological replicates separately. First, adapter and quality trimming of reads was performed using Trimmomatic; Cutadapt [11] was then used to remove all the adapter sequences that were not trimmed in the first phase. Trimmed reads were aligned to GRCm38 using Bowtie [12] with parameters -a -m 1 --best --strata. Duplicate reads, which could represent PCR artifacts, were removed using MarkDuplicates from Picard (picard.sourceforge.net/command-line-overview.shtml). Tools from the Pyicoteo suite [13] were used to call CLIP-Seq peaks. First, all reads were extended to a length of 36 nucleotides using the pyicos extend tool. Then, CLIP-Seq peak calling was performed using the Pyicoclip tool. Ensembl 77 GTF file was supplied to generate exploratory regions, using the option --region-magic genebody. For each replicate, only peaks with p-value < 0.001 were retained. Finally, BEDTools merge [14] with option -d 50 was used to merge peaks from all the replicates; all those clusters that resulted from the merging peaks identified in at least two replicates were taken as FUS binding sites. Transcripts bound by FUS in the 3’UTR were found by intersecting the genomic coordinates of these untranslated regions with those of FUS peaks using BEDTools intersect.

1. Bolger AM, Lohse M, Usadel B (2014) Trimmomatic: a flexible trimmer for Illumina sequence data. Bioinformatics 30:2114–2120. doi: 10.1093/bioinformatics/btu170

2. Flicek P, Amode MR, Barrell D, et al (2014) Ensembl 2014. Nucleic Acids Research 42:D749–55. doi: 10.1093/nar/gkt1196

3. Li H, Durbin R (2010) Fast and accurate long-read alignment with Burrows?Wheeler transform. Bioinformatics 26:589–595. doi: 10.1093/bioinformatics/btp698

4. Kim D, Pertea G, Trapnell C, et al (2013) TopHat2: accurate alignment of transcriptomes in the presence of insertions, deletions and gene fusions. Genome Biol 14:R36. doi: 10.1186/gb-2013-14-4-r36

5. Li H, Handsaker B, Wysoker A, et al (2009) The Sequence Alignment/Map format and SAMtools. Bioinformatics 25:2078–2079. doi: 10.1093/bioinformatics/btp352

6. C T, DG H, M S, et al (2012) Differential analysis of gene regulation at transcript resolution with RNA-seq. Nature Biotechnology 31:46–53. doi: 10.1038/nbt.2450

7. Zhao S, Guo Y, Sheng Q, Shyr Y (2014) Advanced heat map and clustering analysis using heatmap3. Biomed Res Int 2014:986048–6. doi: 10.1155/2014/986048

8. Muller H, Marzi MJ, Nicassio F (2014) IsomiRage: From Functional Classification to Differential Expression of miRNA Isoforms. Frontiers in Bioengineering and Biotechnology 2:475. doi: 10.3389/fbioe.2014.00038

9. Risso D, Schwartz K, Sherlock G, Dudoit S (2011) GC-Content Normalization for RNA-Seq Data. BMC Bioinformatics 12:480. doi: 10.1186/1471-2105-12-480

10. Robinson MD, McCarthy DJ, Smyth GK (2009) edgeR: a Bioconductor package for differential expression analysis of digital gene expression data. Bioinformatics 26:139–140. doi: 10.1093/bioinformatics/btp616

11. Martin M (2011) Cutadapt removes adapter sequences from high-throughput sequencing reads. EMBnetjournal 17:10. doi: 10.14806/ej.17.1.200

12. Langmead B, Trapnell C, Pop M, Salzberg SL (2009) Ultrafast and memory-efficient alignment of short DNA sequences to the human genome. Genome Biol 10:R25. doi: 10.1186/gb-2009-10-3-r25

13. Althammer S, Gonz lez-Vallinas J, Ballar C, et al (2011) Pyicos: a versatile toolkit for the analysis of high-throughput sequencing data. Bioinformatics 27:3333–3340. doi: 10.1093/bioinformatics/btr570

14. Quinlan AR, Hall IM (2010) BEDTools: a flexible suite of utilities for comparing genomic features. Bioinformatics 26:841–842. doi: 10.1093/bioinformatics/btq033
