# Supplementary material for: A Regulatory Circuitry Between Gria2, miR-409, and miR-495 Is Affected by ALS FUS Mutation in ESC-Derived Motor Neurons
Source: Mol Neurobiol. 2018 Feb 12;55(10):7635–51. doi: 10.1007/s12035-018-0884-4 (PMC6132778; doi:10.1007/s12035-018-0884-4)

Online resource

Molecular Neurobiology

A regulatory circuitry between Gria2, miR-409 and miR-495 is affected by ALS FUS mutation in ESC-derived motor neurons

Davide Capauto[1,2], Alessio Colantoni[2], Lei Lu[3], Tiziana Santini[1], Giovanna Peruzzi[1], Silvia Biscarini[1,2],, Mariangela Morlando[2], Neil A. Shneider[3], Elisa Caffarelli[4], Pietro Laneve[1,*], Irene Bozzoni[1,2,4,5,*].

1. Center for Life Nano Science@Sapienza, Istituto Italiano di Tecnologia, Rome, Italy.
2. Department of Biology and Biotechnology, Sapienza University of Rome, Italy.
3. Department of Neurology, Center for Motor Neuron Biology and Disease, Columbia University, New York City, NY, USA.
4. Institute of Molecular Biology and Pathology, CNR, Rome, Italy.
5. Institute Pasteur Fondazione Cenci-Bolognetti, Sapienza University of Rome, Italy.

[*] Corresponding authors:
e-mail: irene.bozzoni@uniroma1.it ; pietro.laneve@iit.it

Supplementary Figure S1

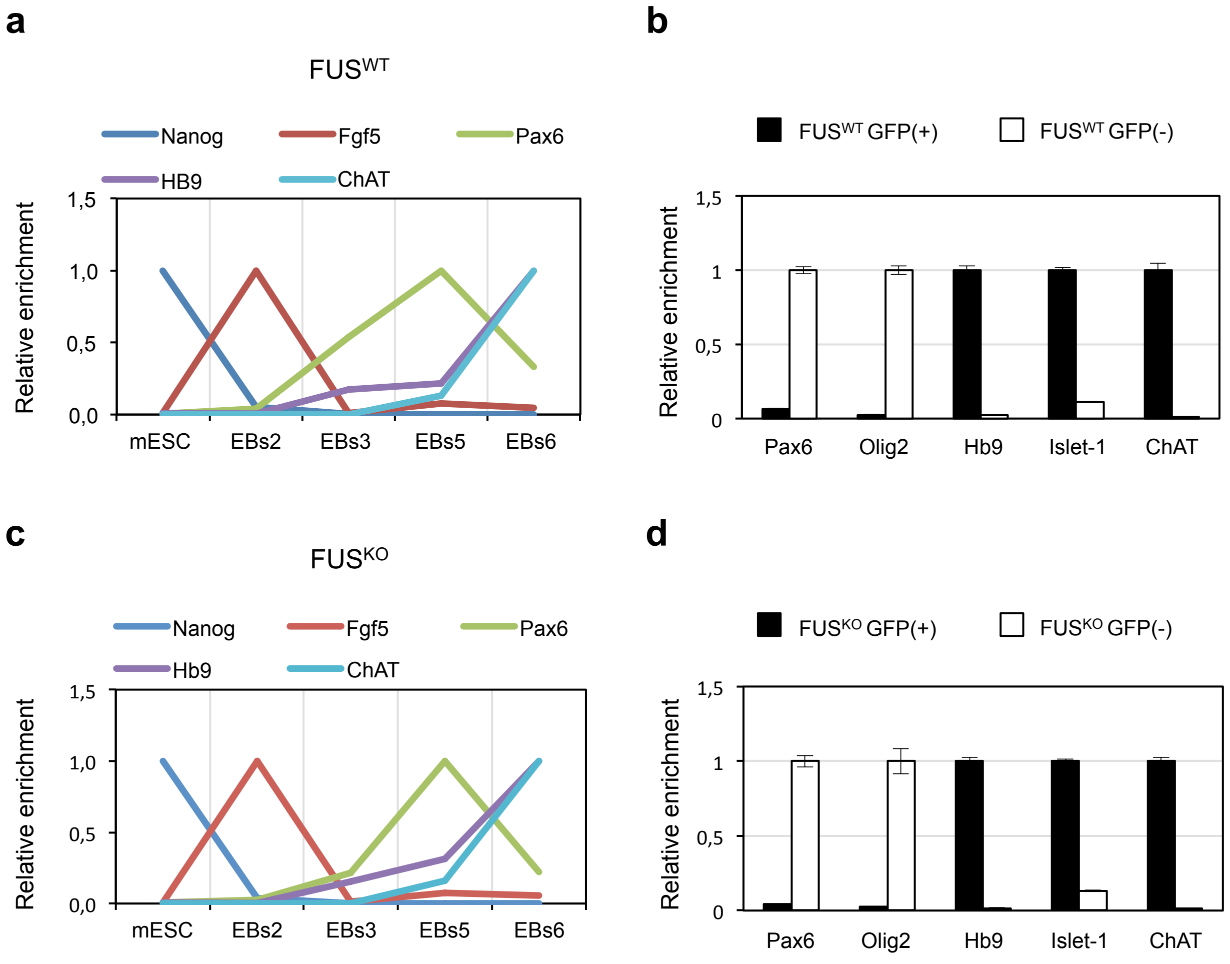

Supplementary Figure S2

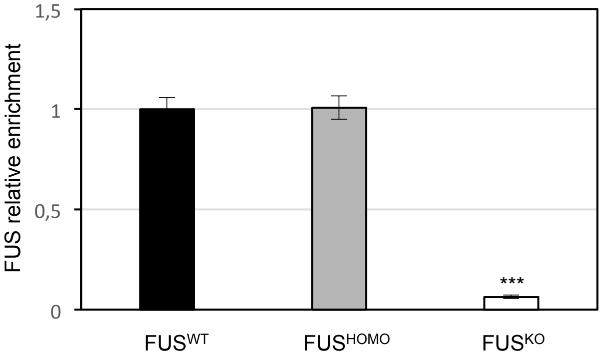

Supplementary Figure S3

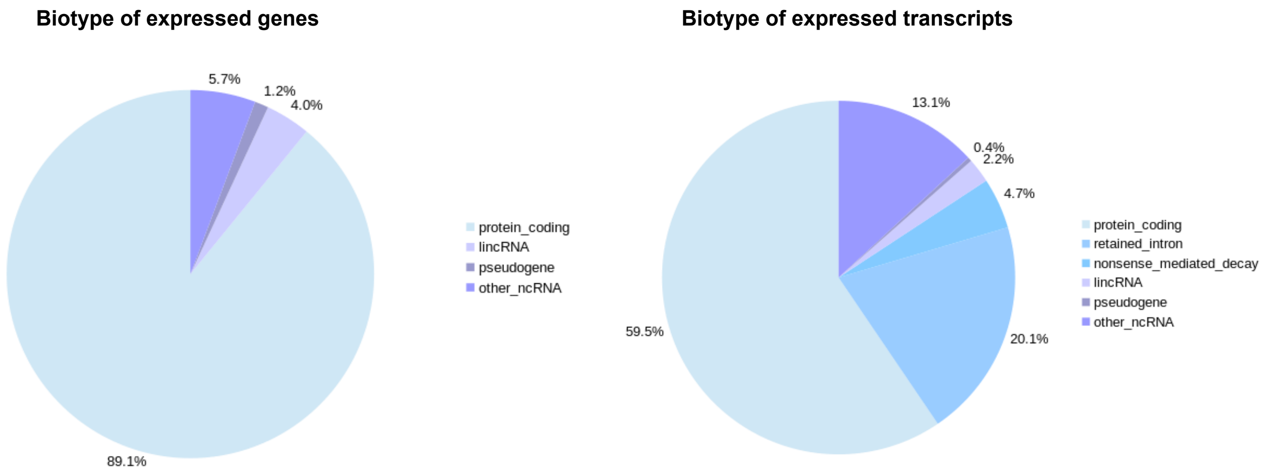

Supplementary Figure S4

Biological process

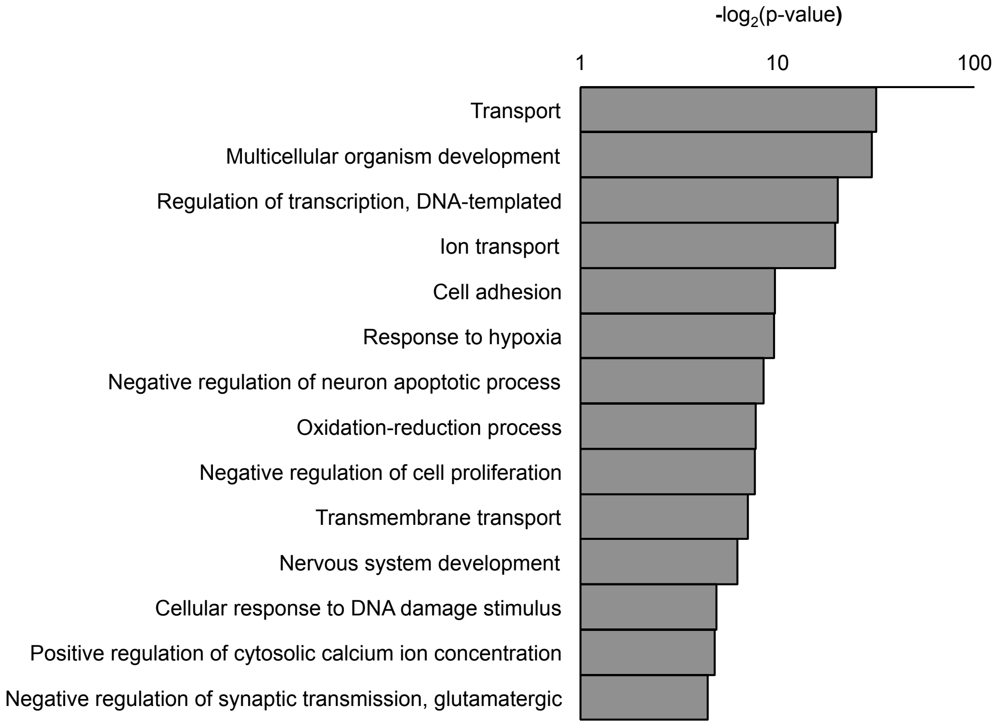

Kegg pathways

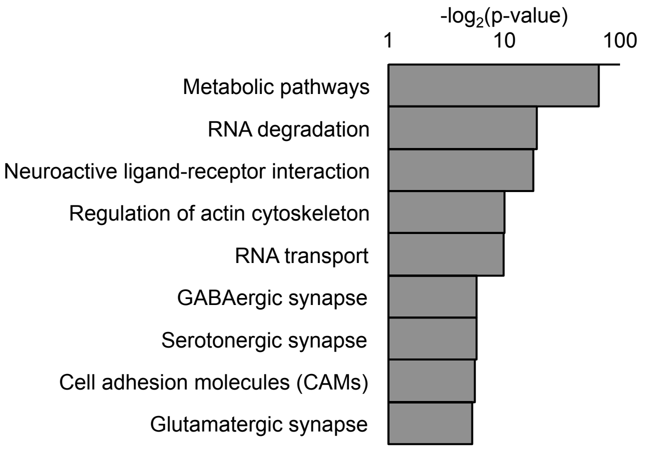

Supplementary Figure S5

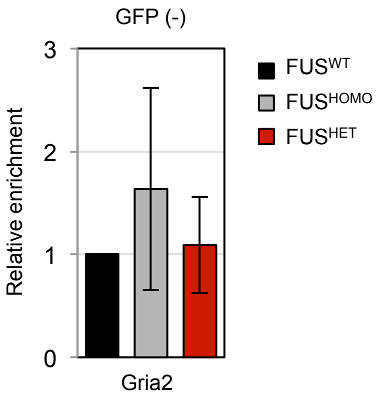

Supplementary Figure S6

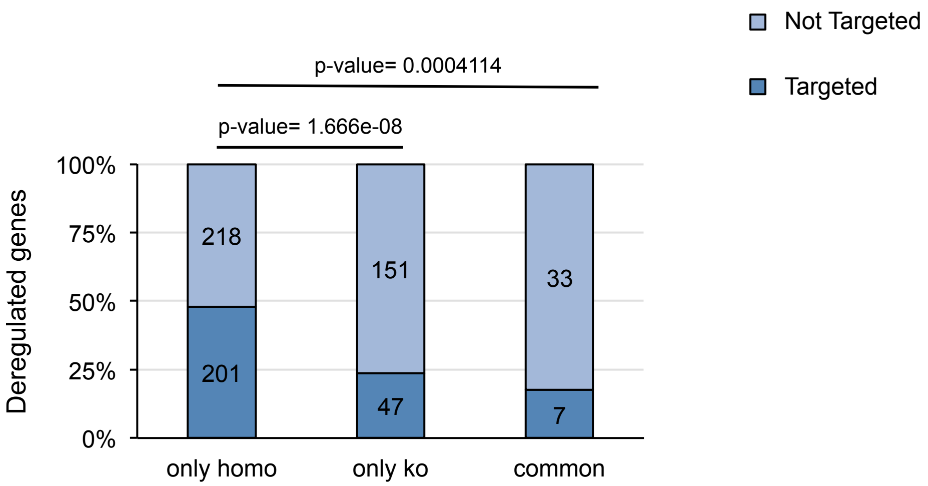

Supplementary Figure S7

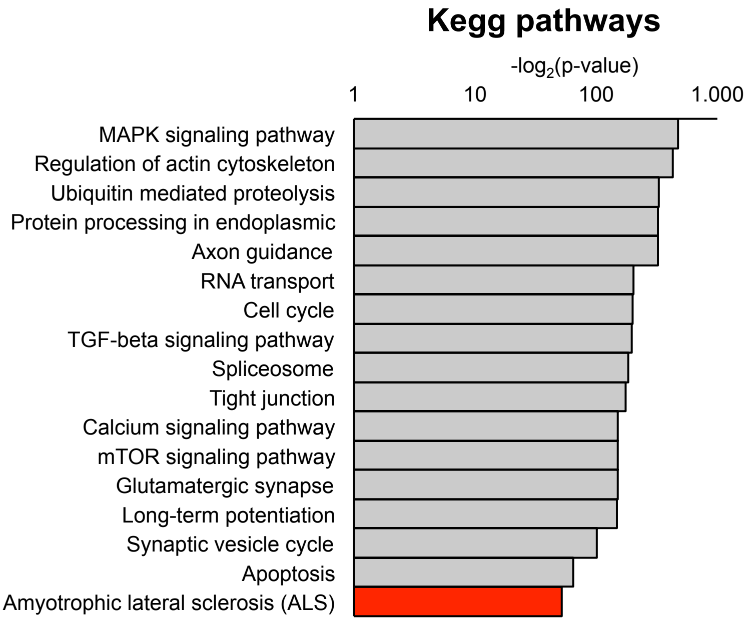

Supplement: Supplementary file 3 — (PDF 140 kb) [file 12035_2018_884_MOESM3_ESM.pdf]
