# Supplementary material for: A Regulatory Circuitry Between Gria2, miR-409, and miR-495 Is Affected by ALS FUS Mutation in ESC-Derived Motor Neurons
Source: Mol Neurobiol. 2018 Feb 12;55(10):7635–51. doi: 10.1007/s12035-018-0884-4 (PMC6132778; doi:10.1007/s12035-018-0884-4)
Supplement: Supplementary file 10 — (DOCX 21 kb) [file 12035_2018_884_MOESM10_ESM.docx]

**Supplementary Figure Legends**

**Supplementary Fig. 1**

**Marker expression during differentiation of FUS^WT^ and FUS^KO^ mESCs into spinal MNs. Related to Fig. 1b and Fig. 1d**

**a.** qRT-PCR profiling of stemness, primitive ectoderm and neural/MN markers along differentiation of FUS^WT^ mESCs to MNs. Cell types/differentiation days (2 to 6) are indicated on the x-axis. For each marker analyzed (indicated above), expression peak is set as 1. Results are expressed in arbitrary units, relative to Atp5o as internal standard.

**b.** qRT-PCR analysis of neural/MN progenitors and MN markers in sorted FUS^WT^ GFP(+) (black bars) and GFP(-) cell populations (white bars). For each marker analyzed (indicated above), expression peak is set as 1. Results are expressed in arbitrary units, relative to Atp5o as internal standard.

**c.** Marker expression during differentiation of FUS^KO^ MNs. Details as in **a**.

**d**. qRT-PCR analysis of neural/MN progenitors and MN markers in sorted FUS^KO^ GFP(+) and GFP(-) cell populations. Details as in **b**.

**Supplementary Fig. 2**

**FUS expression in FUS^WT^, FUS^HOMO^ and FUS^KO^ MNs. Related to Fig. 2**

qRT-PCR analysis of FUS mRNA in FUS^WT^ (black), FUS^HOMO^ (grey) and FUS^KO^ (white) in sorted MNs. Expression in FUS^WT^ was set as 1. Results (+/- SEM) from three biological replicates are expressed in arbitrary units, relative to Atp5o as internal standard.

**Supplementary Fig. 3**

**Pie chart representing biotype abundances for the genes and transcripts identified by RNA-Seq in mouse MNs (minimum FPKM > 0.1). Related to Fig. 3**

**Supplementary Fig. 4**

**Gene ontology analysis of FUS^KO^ deregulated genes. Related to Fig. 3d**

Representative functional categories of genes differentially expressed between FUS^KO^ and FUS^WT^ according to Gene Ontology analysis. Biological Processes (upper diagram) or Kegg Pathways (lower diagram) are shown.

**Supplementary Fig. 5**

**RNA expression analysis of Gria2 in FUS mutant GFP(-) cells. Related to Fig. 3e**

qRT-PCR analysis of Gria2 in FUS^HOMO^ (grey bar) and FUS^HET^ (red bar) GFP(-) cells compared to FUS^WT^ (black bar), set as 1. Results (means +/-SEM) from three biological replicates are expressed in arbitrary units and are normalized to the mean value of Atp5o mRNA.

**Supplementary Fig. 6**

**Fraction of deregulated genes targeted by miRNAs in different FUS contexts. Related to Fig. 5a**

Percentage of genes deregulated exclusively in FUS^HOMO^, or in FUS^KO^ or in both conditions, which are putatively targeted by miRNAs altered in the opposing direction.

**Supplementary Fig. 7**

**Gene ontology analysis of predicted target genes of FUS^HOMO^ deregulated miRNAs. Related to Fig. 3**

Representative functional categories of genes putatively targeted by microRNAs differentially expressed between FUS^WT^ and FUS^HOMO^ MNs. Kegg Pathways analysis is reported in the diagram. ALS gene category is highlighted in red.
